# Entrance Qualifications

**Published:** 1899-11

**Authors:** 


### Editorial.

### ENTRANCE QUALIFICATIONS.

Upon the "Original" pages and in "Reports of Societies" in this number our readers will find a paper by Professor Darby on this subject, and the discussion following it at one of the meetings of the Academy of Dental Science, Boston. This question is now agitating the professional dental mind in this country as never before. It is by no means a new question, for it has been considered in a fragmentary way for the past thirty or more years, and has received some attention upon the pages of this journal. The subject has, however, acquired added importance through the action, year by year, of the National Association of Dental Faculties, and through the generally unwise pressure brought to bear upon that body and the colleges of the United States by State boards and by the National Association of Dental Examiners.

The question is one of supreme importance, and while no new light may have been thrown upon it at Boston, it is well that it has been given consideration in that acknowledged centre of intelligence.

It is quite evident, from the remarks made at that meeting, that the social standing was considered by some there of vastly more importance than skill in practice. This seems to be manifest in the remarks of Dr. Smith, Dean of Harvard's Department of Dentistry. He says, "I do not assent that it [dentistry] is equal to the position occupied by other professions, notably law, medicine, and divinity, because it lacks the preliminary intellectual training. Dental schools should be graduate schools, and require a degree of letters, or its equivalent, to enter upon the study of dentistry."

It is true that dentistry does not hold the same place in public estimation that law, medicine, and divinity do, but issue must be taken with the opinion of Dean Smith when he asserts that this is due to a lack of "preliminary intellectual training." It is safe to assume that, were all the members of the dental profession in practice to-day possessed of the A.B. or even the much coveted Ph.D. degree, this opposition to dentists would not be changed in the least, and the reason for this is not far to seek. The three conditions that are extremely distasteful to the average social critic are extreme youth, work, and a lack of family inheritance. This may be explained by the fact that youth is disagreeable, work is degrading, and he or she who has not received the stamp of ancestry of a certain kind is not to be regarded as fitted for a place in the social life of the world. In intellectual centres cultivation will take the place of the latter condition, and the man or woman who makes a name in either of the three professions will be accepted, but all the more will he or she be recognized if there be added to this a name renowned in the past family history. It is not necessary to dispute these conditions, for they exist everywhere and are part of the life of individuals and communities in exact proportion to the intellectual standing or the bank account.

The one crime of the dentist is that he is in a profession without age. In other words, he is of modern growth. In the second place, he works with his hands, and this cannot be forgiven; and should he be fortunate to have had distinguished ancestors, he has become a degenerate because he has descended from the high estate of his forefathers.

It may be unkind to those who are anxious to be received upon the same equality with the professions named to say that they need never expect to have the same standing as these as long as the unrighteous standard which now prevails is in force.

Many entertain the opinion expressed by Dean Smith, that a higher preliminary training will make better dentists. Within reasonable limits this is true, but it has its limitations, and if it should ever happen that dentistry should reach the standard, set up by some, of the A.B. degree, then most certainly will the practical side of our profession be in a worse condition than it is at present. Cases might be cited where men have taken the highest degrees and succeeded subsequently in mechanical pursuits. These are the exceptions observed in all general laws, but they in nowise weaken the general proposition that in proportion to years spent in study will the manual dexterity be lost; and no one will dispute the fact that to secure the A.B. degree, as generally understood, means the best and formative years of a young man's life.

Our English brethren have been unduly sensitive over the expression "American dentistry." They seem to have regarded the term as in common use in this country, but such is not the fact. No intelligent person on this side of the water ever imagines, much less arrogantly speaks of, the work done in this country as superior to that abroad. The term originated on the Continent from the fact that American practitioners there in the earlier days pursued methods peculiar to practice here, and hence the distinguishing name applied not by themselves, but others.

While this explanation is the true one, can it not be said that there is an American dentistry that upon its practical side has been superior to that of older civilizations? Without egotistic assumption, it may be stated that nearly all the improvements in dental and operative mechanics have had their origin in the United States. Allusion need only be made to a few of these: the dental engine, the mallets, electrical and mechanical, rubber and celluloid plates, so-called continuous gum, crown- and bridge-work, and the rubber dam. The list might be extended, but these typify the important advances made.

In Europe, including Great Britain, there has been a marked advance in theoretical knowledge, and we on this side are not slow to acknowledge our indebtedness for the work accomplished; but the observer will find that those things that make progress in a practical sense have had, with few exceptions, their origin in America. So that there is an American dentistry, or rather a mental activity, here that is never satisfied with things as they are, and this, it is assumed, has a larger development in this country than in any other of our modern civilizations. Space will not permit the writer to enter into the reasons for this, but this activity exists as part of our inheritance.

The thought that follows this statement brings the writer to the consideration of the question, Was this accomplished by men who possessed the preliminary qualifications considered so necessary in Europe? The answer must be in the negative. The higher training may lead to a quicker perception of things, as in the case of Arthur, the scholar, who observed the cohesive effect of pure gold when in sheet form it was brought in contact. Its introduction resulted in a revolution in practice. The inventive mind coupled with mechanical tastes may produce positive results in a highly trained intellect, but these are exceptional, and must not be regarded as proving the special value of the higher training desired. In the world of dental mechanics the scholarly man makes but a poor exhibit, while the work of the ordinary mechanic has become the common property of the world.

The life-work of Dr. Bonwill, recently deceased, accentuates this very fully. Had he been forced to extend his preliminary training, it might have been of great advantage as a personal acquirement, but it certainly would have detracted from his ability as a mechanic, so markedly manifested to the advantage of the dental profession.

The writer would not be understood as advocating a low standard of qualification. No one entertains a higher estimate of an education, thorough in every respect, than he, but he appreciates the fact that dentistry is peculiar in that it is a mixed profession, combining mechanics and the collateral scientific branches so thoroughly that to excel in it a different course must be pursued in the training from that adopted by other professions.

The idea thrown out in the discussion, to which allusion has been made, that a degree from a technical school would be the best educational foundation, as the Institute of Technology, coincides with the writer's views. The manual training schools should be the feeders of dental colleges, and should President Eliot's ideas be carried out, there could then be no objection to the A.B. degree, for the young man would enter dentistry properly equipped to meet all the difficulties of special training and the contingencies of practice.

